# Control of Whole Heart Geometry by Intramyocardial Mechano-Feedback: A Model Study

**DOI:** 10.1371/journal.pcbi.1002369

**Published:** 2012-02-09

**Authors:** Theo Arts, Joost Lumens, Wilco Kroon, Tammo Delhaas

**Affiliations:** Department of Biomedical Engineering, Cardiovascular Research Institute Maastricht, Maastricht University, Maastricht, The Netherlands; University of California San Diego, United States of America

## Abstract

Geometry of the heart adapts to mechanical load, imposed by pressures and volumes of the cavities. We regarded preservation of cardiac geometry as a homeostatic control system. The control loop was simulated by a chain of models, starting with geometry of the cardiac walls, sequentially simulating circulation hemodynamics, myofiber stress and strain in the walls, transfer of mechano-sensed signals to structural changes of the myocardium, and finalized by calculation of resulting changes in cardiac wall geometry. Instead of modeling detailed mechano-transductive pathways and their interconnections, we used principles of control theory to find optimal transfer functions, representing the overall biological responses to mechanical signals. As biological responses we regarded tissue mass, extent of contractile myocyte structure and extent of the extra-cellular matrix. Mechano-structural stimulus-response characteristics were considered to be the same for atrial and ventricular tissue. Simulation of adaptation to self-generated hemodynamic load rendered physiologic geometry of all cardiac cavities automatically. Adaptation of geometry to chronic hypertension and volume load appeared also physiologic. Different combinations of mechano-sensors satisfied the condition that control of geometry is stable. Thus, we expect that for various species, evolution may have selected different solutions for mechano-adaptation.

## Author Summary

The heart is known to adapt size of the cavities and thickness of the walls to the pumping requirements set by blood pressure and blood flow. We think that mechanical load of the cardiac tissue provides feedback signals for adaptation of mass and thickness of the cardiac walls. Many cellular mechanisms are known where mechanical load initiates a cascade of chemical reactions, eventually affecting structure and mass of the tissue. Because these mechanisms interact intricately, understanding of the system of adaptation as a whole is tremendously complicated. We present a novel approach by considering adaptation as a control system. Using the principle that control should converge to a stable end state, general rules are found that should be satisfied on transfer of mechanical load to structural adaptation in the cells of the tissue. We think that deeper understanding of the mechanism of adaptation requires that knowledge on mechano-transductive pathways is placed in the context of regarding adaptation as a system. Knowledge on adaptation of cardiac geometry to mechanical load is crucial in predicting long term effects of pathologic disorders or therapeutic interventions that chronically affect blood pressure or blood flow.

## Introduction

Mass and area of the cardiac walls vary with changing mechanical load. We regard development and preservation of cardiac geometry as a result of homeostatic control that keeps mechanical load of the cardiac tissue in the physiologic range. Following this principle, we think that cellular mechanisms sense mechanical load imposed by pressure and volume in the cavities of the heart. Obtained information about this load is used to change tissue mass and structure locally. Subsequently, macroscopic geometry of the cardiac cavities and walls changes, thus affecting circulation hemodynamics. As a result, mechanical load of the cardiac tissues changes, implying closure of the control loop for adaptation of cardiac geometry.

**Figure 1:**
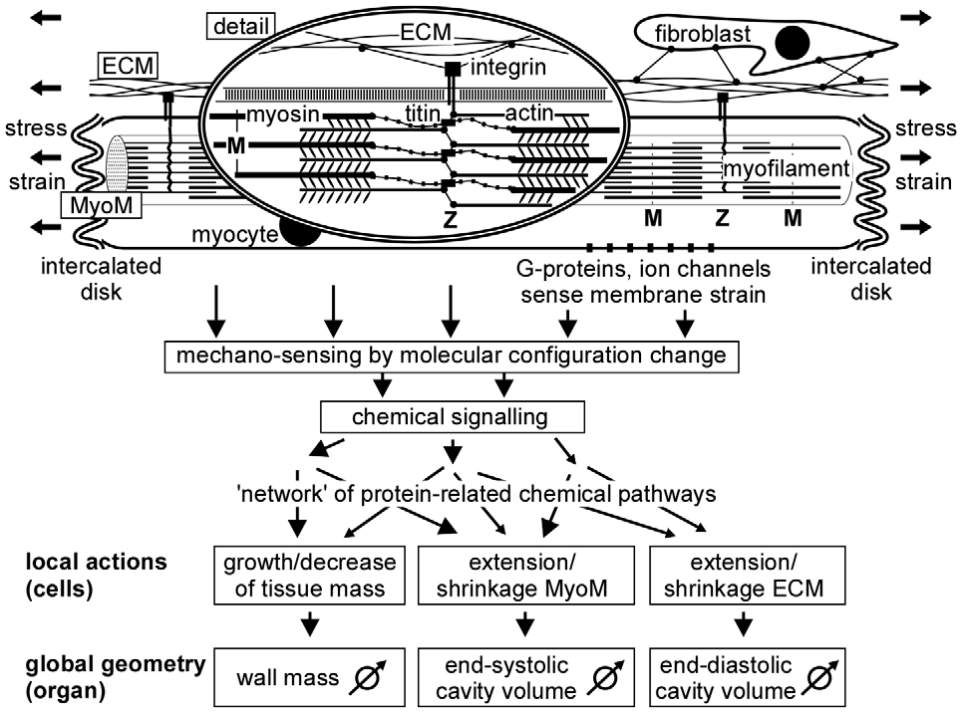
Schematic of adaptation of wall geometry to mechanical load. Global stress and strain of the tissue determine forces and deformation of the myocyte matrix (MyoM) and extracellular matrix (ECM). Within the myocyte, axial forces are born by the sarcomeres in the myofilaments, consisting of actin and myosin, mutually coupled by cross-bridges. The Z-disk connects actin and titin of neighboring sarcomeres. The distance between Z-discs represents sarcomere length, ranging from 1.7 to 2.3 µm. Titin parallels actin, and is connected to myosin. The Z-disks are connected transversely to the ECM by integrins, traversing the cell membrane of the myocyte. Mechano-sensing has been reported around the Z-disk, within the cell membrane of the myocyte and in the intercalated disks, forming axial connections between myocytes. Fibroblasts are also mechano-sensitive. Resulting chemical signals follow a network of intertwined pathways of chemical activity, resulting in local actions of adaptation. Summed effects of local actions determine global changes in geometry of the myocardial wall.

Several mechano-sensing structures have been identified in cardiac tissue (Fig. 1) [1], [2], [3], [4]. The major part of cardiac mass consists of myocytes, being the cells that are responsible for cardiac contraction. Myocytes contain myofilaments, which are composed of a serial repetition of sarcomeres, representing the basic contractile units. Sarcomere length decreases during contraction from about 2.2 µm to 1.8 µm by sliding of the thin actin filaments past the thick myosin filaments. In the sarcomere, the Z-disc forms a planar network of cross-connections between the actin filaments. The tips of the myosin filaments are connected to the Z-disk by strands of titin parallel to the actin filaments. The Z-discs extend over the different filaments in the cell and across the cell membrane with integrin connections to the extracellular matrix (ECM). The integrins respond to stress between myocyte and ECM by activating protein-mediated chemical pathways, leading to structural changes of the tissue [5], [6], [7]. Connections of actin and titin to the Z-disk are mechano-transductive too [4], [8], [9], [10]. G-protein coupled receptors appear sensitive to strain of the cellular membrane [2], [11]. Stretch-activated ion channels modulate intracellular Ca-concentration [12], [13], [14], [15]. Tissue stretch elevates the number of fibroblasts and formation of ECM-related proteins [16], possibly leading to fibrosis.

**Figure 2:**
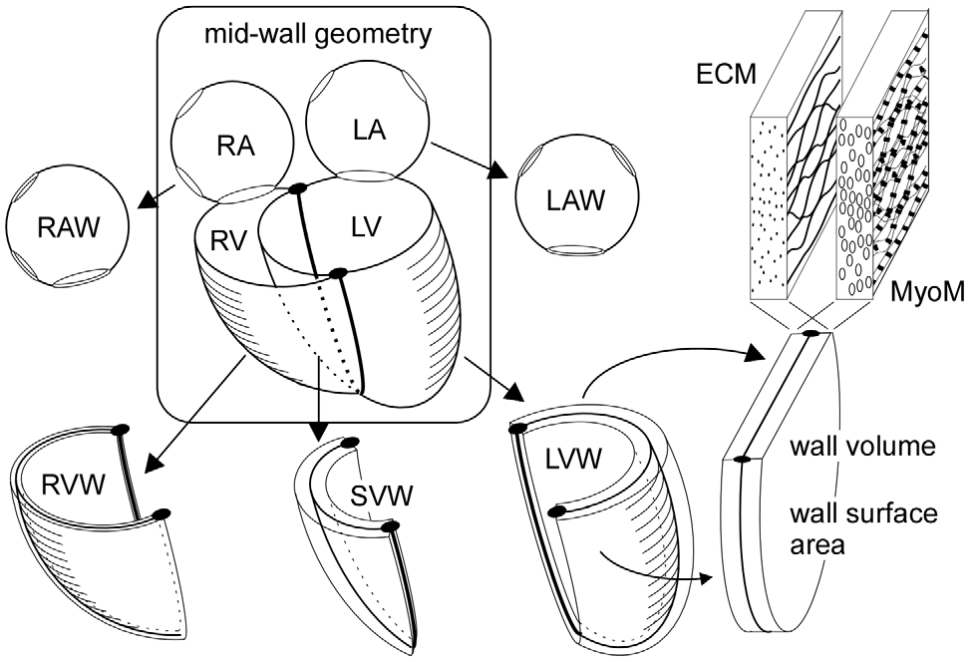
Composition of the heart by 5 walls with MyoM and ECM. Curved mid-wall surface form the core of macroscopic geometry of atria and ventricles. Left and right atrium (LA, RA) are enclosed by single curved walls (LAW, RAW). The ventricular unit is composed of left, septal and right ventricular wall (LVW, SVW and RVW), enclosing left and right ventricle (LV, RV). Geometry of each wall is deducted from unfolding the wall to a flat surface. Each wall has a volume and a mid-wall area. A wall contains a myocyte matrix (MyoM) intertwined with the extracellular matrix (ECM). Both matrices have their own reference mid-wall area, linking their ultrastructure to the macroscopic geometry of the wall.

Mechano-transductive signals modify tissue structure, leading to change of macroscopic cardiac geometry. In Fig. 2, the configuration of mid-wall surfaces forms the core of cardiac geometry. We consider the heart to be composed of four cavities enclosed by five walls. Both atrial cavities are enclosed by curved walls with holes for valves and blood vessel connections. For simplicity we neglected the atrial septum. The ventricular unit is composed of three curved walls, enclosing two cavities. The openings at the base are covered by a non-contractile basal sheet, harboring the four cardiac valves. For each wall, volume and mid-wall area are defined by unfolding the wall to a flat surface. In this representation, wall thickness equals wall volume divided by wall area.

Micro-structurally, a cardiac wall is composed of a network of connected myocytes and a network of connective tissue (Fig. 2). For the latter networks we use the terms myocyte matrix (MyoM) and extra-cellular matrix (ECM), respectively. With a change of cavity volume the size of matrices changes with that of the walls. We need an objective indication of the macroscopic size of these matrices relative to ultra-structural features. Thus, the reference size of the MyoM in a wall is defined as the area of the mid-wall surface, when having stretched the sarcomeres in the myocytes to a length of 2.3 µm. For the ECM, in absence of clearly visible ultra-structural length markers, the reference size is referred to the strain level at which ECM stress equals the target value of adaptation.

Cardiac wall function is determined mainly by three parameters, i.e., wall volume, reference area of the MyoM and that of the ECM, respectively. Considering the myocardial tissue to be healthy and working optimally, wall volume is proportional with the maximum amount of contractile work that can be delivered. The reference area of the contractile myocyte matrix MyoM determines enclosed cavity volume at which sarcomere length is optimal for power generation. The reference area of the ECM determines the diastolic pressure-volume relation. An increase of this area causes a shift of this relation towards higher volumes.

Within a single cardiac wall, reference areas for MyoM and ECM are generally different. For the ventricles, volume is maximal at end-diastole, after which the ventricles contract immediately. So, for ventricular walls, ECM and MyoM reference areas are similar. In the atria, volume is maximal at the beginning of ventricular diastole. In the subsequent ventricular fast filling phase, the atria empty. Next, the atria are activated, causing further emptying of the atria. So, for atrial walls, maximum wall area is considerably larger than wall area at the beginning of atrial contraction. Thus, in contrast with ventricular walls, for the atrial walls ECM reference area is considerably larger than MyoM reference area.

With eccentric hypertrophy, both wall mass and MyoM wall area are elevated, resulting in proportional increase of wall thickness and cavity diameter [17], [18], [19], [20]. With concentric hypertrophy, wall mass is elevated, but MyoM wall area remains about the same, thus causing the wall to thicken while cavity diameter is maintained [21]. ECM reference wall area is a major determinant of diastolic filling. With diastolic dysfunction, ECM area is small relative to MyoM area, implying that stiffness of passively elastic structures in the wall hamper proper diastolic filling. As a result, in the end-diastolic state, the sarcomeres of the MyoM are not sufficiently lengthened for a forceful contractile stroke.

For proper adaptation of cardiac wall geometry to mechanical load, information gathered by the various mechano-sensors in that wall is processed to a well-tuned blend of structural changes (Fig. 1). Specific sensors detect specific types of mechanical load by altering the configuration of mechano-sensitive molecules, thus initiating cascades of chemical reactions. The various pathways of these reactions mutually overlap and interact [1], [3], [22], thus forming a complex network of information processing. The output of this network determines changes in myocardial structure, macroscopically leading to adaptation of wall mass, MyoM area and ECM area. It is not clear yet, how the different mechano-sensed signals contribute to the various types of structural adaptation.

The present computational modeling study focuses on how mechano-sensed signals affect tissue structure, and thereby determining global cardiac geometry. We used a novel approach by considering general principles of control systems theory. A first requirement is stability of homeostatic control of cardiac geometry, i.e., perturbations in mechanical or hemodynamic load must be properly compensated. Secondly, the system should take care for the myocytes to operate in a range of sarcomere length and mechanical load that is optimal for mechanical performance.

## Models

### The CircAdapt model

**Figure 3:**
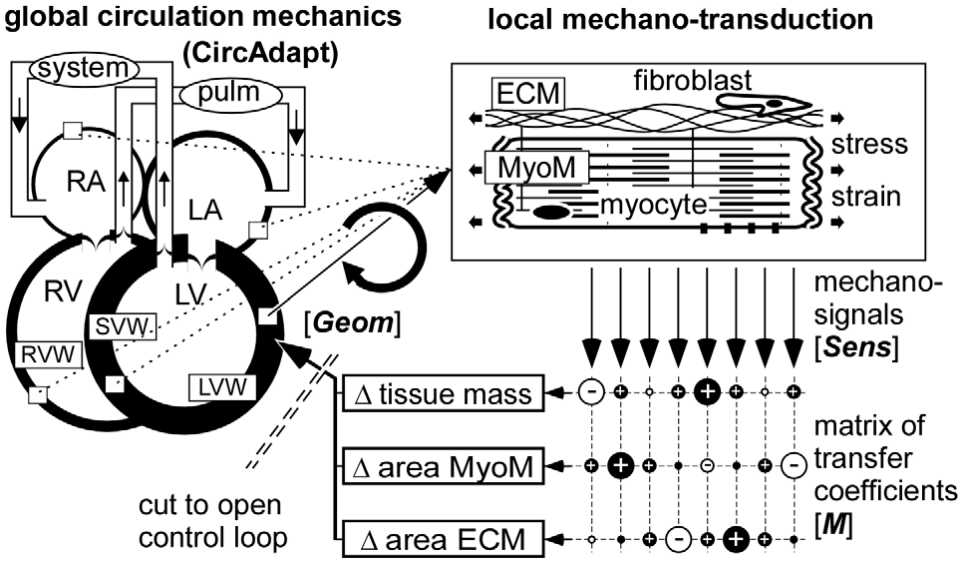
Modeling control of myocardial wall geometry. In the CircAdapt model of the whole circulation (left), geometry of the walls determines hemodynamic performance, which in turn determines mechanical load of the wall material (upper right). Local mechano-sensing invokes changes in tissue structure (lower mid) and geometry, by which the control loop for adaptation of wall geometry is closed. A matrix of transfer coefficients (lower right) is used to estimate changes in tissue structure, causing geometry to adapt. Symbols [*Sens*, *M*, *Geom*] are used in Eq. (2–9). Abbreviations are explained in the general list.

The CircAdapt model (Fig. 3, upper left) simulates beat-to-beat hemodynamics and mechanics of the closed circulation [23]. The source code and short manual are added as supporting files. In this model, the left (LA) and right (RA) atrial cavities of the heart are encapsulated by their respective left (LAW) and right (RAW) atrial walls. The left (LV) and right ventricular (RV) cavities are encapsulated by the left (LVW) and right (RVW) ventricular lateral walls, and separated by the ventricular septum (SVW). Ventricular interaction (TriSeg model) is derived from the equilibrium of forces in the three walls, acting on their common junction [24]. As seen from the heart, the large arteries are simulated by a non-linear representation of the arterial characteristic impedance in series with an arterial compliance [25], [26]. The large veins are represented similarly, as seen from the atria, consisting of a venous characteristic impedance and a venous compliance. The systemic peripheral resistance connects the systemic arterial compliance with the systemic venous compliance. Similarly, the non-linear pulmonary peripheral resistance connects pulmonary arterial and venous compliances. Myofiber stress depends on myofiber strain, strain rate and time, as reported in experiments on isolated cardiac muscle fibers [27], [28]. A unique and useful feature of the CircAdapt model is that the walls of heart and blood vessels can be shaped by adaptation signals, derived from mechanical load of the constituting tissues.

The core of the CircAdapt model is a set of differential equations with about 30 state variables, being 8 volumes (arteries and veins of left and right circulation and 4 heart cavities), 6 inertias (4 valves and 2 atrial inlet ducts), 5 contractilities and 5 sarcomere lengths (5 cardiac walls), and 2 geometric measures related to ventricular interaction. Starting with some reasonable initial state, about 30 beats (time resolution 2 ms) are simulated to reach steady state. In that steady state, hemodynamics of moderate exercise (Table 1) is satisfied. Blood pressure is controlled by adjustment of circulating blood volume. This steady state is used as reference state for the simulations. The model is simulated in Matlab (MathWorks, www.mathworks.com). Calculation time is 4 seconds per heart beat on a HP-Pavilion laptop computer (www.hp.com). The source code is added as supplementary file.

**Table 1:** Myocardial wall geometry and hemodynamics.

*wall geometry*	LAW	RAW	LVW	SVW	RVW
wall volume [ml]	13.6	3.0	99.0	48.0	43.6
MyoM wall area [cm^2^]*	78.3	56.3	117.6	74.3	161.9
ECM wall area [cm^2^]	105.6	80.0	113.7	71.2	153.1
***cavity volumes***	LA	RA	LV	RV	
	*exercise(rest)*				
maximum [ml]	114 (75)	82 (56)	186 (143)	150 (107)	
minimum [ml]	32 (24)	20 (19)	77 (70)	44 (35)	
***hemodynamics***			*exercise(rest)*		
heart rate	[beats/min]		141 (71)		
mean arterial pressure	[mmHg]		92 (92)		
mean pulm. pressure	[mmHg]		21.4 (14.9)		
mean aortic flow	[l/min]		15.3 (5.1)		

LAW, RAW, LVW, SVW, RVW: see Fig. 2.

*referred to sarcomere length = 2.3 µm.

### Model of mechano-sensing

The CircAdapt model is used to calculate myofiber stress and strain in the myocardial walls, given their geometry (Fig. 3). We investigated by simulation how local tissue structure responds to local changes in mechano-sensed variables. A set of candidate variables has been proposed based on known physiological mechanisms of mechanotransduction. Below we show how these variables are calculated from available stress and strain signals.

Within the tissue, total stress *S_tot_* along the fiber direction is a summation of stress in the various substructures. Stress *S_act_* is generated by the activated cross-bridges, connecting actin and myosin. This stress depends on the degree of activation which is a function of sarcomere length *L_s_* and time *t* after activation. Stress *S_tit_* is born by the passive elastic structures inside the myocyte such as titin. By neglecting viscous effects, this stress depends directly on *L_s_*. Stress *S_ecm_* is born by the passive elastic structures outside the myocyte. This stress depends on time varying myofiber strain *e_f_*(*t*), and has no direct relationship with sarcomeres inside the myocyte. However, since sarcomeres and the surrounding tissue components share the same deformation field, for mathematical convenience *e_f_*(*t*) is referred to reference sarcomere length *L_s,ref_*. Thus, for total stress *S_tot_* we used
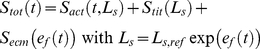
(1)The candidate mechano-sensed variables are listed in Table 2. Variable *S_ecm,max_* represents maximum stress in the ECM during the cardiac cycle. The signal may result from an increasing chance of rupturing collagen struts in the ECM, causing dilation of the ECM directly. Moreover, near locations of rupture, severe deformations may induce mechano-transductive signals through integrins, connecting cell interiors to the ECM. Variable *S_act,max_* indicates maximum stress in the actin filaments, possibly detected by molecules in the Z-disks. The signal is closely linked to contractility. Variable *S_int_* refers to stress between myocyte interior and myocyte exterior, possibly born by the integrins. Integrins are known to connect Z-disks of myocytes to the collagen filaments of the ECM. If either stress in the myocytes or stress in the ECM, or both, is low, stress in the integrin connection is low. Variable *S_Ztit_* refers to stress within the Z-disc between actin and titin filaments. Complex mechano-sensitive molecules are found near the connection sites of these filaments within the Z-disc. Variable *L_s,act_* indicates working sarcomere length during active contraction, obtained by weighing sarcomere length with actin stress. Similarly, *L_s,int_* represents sarcomere length, weighted with stress between cell interior and exterior. Variable *e_act_* represents strain changes during the active state, implemented by weighing with actin stress. Finally, variable *w_stroke_* represents stroke work density.

**Table 2:** Candidate sensed variables.

#	symbol	equation	meaning
1	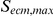	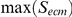	max stress ECM
2	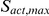	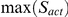	max active stress
3	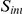	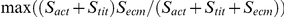	max stress between myocyte and ECM
4	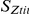	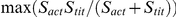	max intra Z-disc stress
5	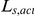	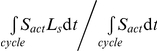	active stress weighted sarcomere length
6	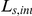	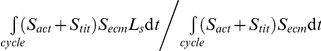	integrin stress weighted sarcomere length
7	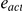	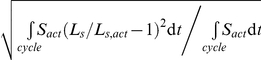	stress weighted strain change
8	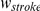	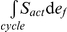	stroke work density

### Model of structural response

Deviations of sensed variables from their target value results in a structural response with a change in geometry so that the sensed variables approach their target value. To facilitate the analysis, the components of vector ***LnSens*** represent the logarithm of calculated sensed variables, stored in vector ***Sens*** (Table 2), normalized to the corresponding target value, stored in vector ***SensRef***:
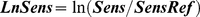
(2)Sensed signals induce structural changes in the myocardial tissue, resulting in change of wall volume *V_wall_*, MyoM wall area *A_myom_* and ECM wall area *A_ecm_*, respectively, as defined in relation to Fig. 2, and forming together the geometric vector ***Geom***. The network of mutually overlapping chemical pathways (Fig. 1) is represented by a currently unknown matrix ***M*** of transfer coefficients (Fig. 3). The change of ***Geom***, induced by the sensed signals, is obtained by the following matrix multiplication:
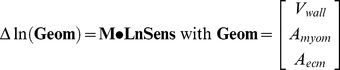
(3)The dot represents matrix multiplication. If ***Sens*** equals ***SensRef***, ***LnSens*** equals zero (Eq. 2), implying no change of ***Geom*** (Eq. 3).

### Model of the control loop

First we consider the open loop relation, in which cardiac geometry determines an adaptive change of tissue structure (Fig. 3). Adaptation is assumed to occur in steps, numbered *t*. Vector ***Geom5*** *_t_* defines geometry of five walls, providing three components each (Eq. 3), thus consisting of 15 components. The addition of number ***5*** indicates handling of all walls together in one vector. The CircAdapt model is used as a function, named CircAdapt, to calculate the associated vector ***Sens5*** *_t_* by:
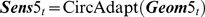
(4)After logarithmic conversion of ***Sens5*** to ***LnSens5*** (Eq. 2), Eq. (3) is used to calculate the increment of vector ln(***Geom5*** *_t_*). Thus, for vector ***Geom5*** *_t_*_+1_ after the *t* ^th^ adaptation step it is found:
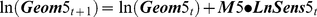
(5)Transfer matrix ***M5*** for all walls together determines feedback properties, and should therefore be optimized for best control characteristics. Assume that vector ***Geom5*** equals ***Geom5Ss*** in steady state. With perfect control, any vector ***Geom5*** *_t_* in Eq. (5), should be followed by vector ***Geom*** *_t+1_* being equal to ***Geom5Ss***, resulting in relation:
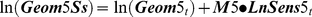
(6)Matrix ***M5*** is solved by choosing a sufficiently large set of geometric vectors ***Geom5*** *_m_* near ***Geom5Ss***, where subscript *m* indicates vector number. Simulations with CircAdapt (Eq. 4) renders the related set of vectors ***Sens5*** *_m_*, resulting with Eq. (2) in vector sets ***LnSens5*** *_m_*. ***M5*** is found by solving Eq.(6):
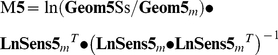
(7)The −1 exponent indicates matrix inversion. Superscript *T* indicates matrix transposition.

### Principle of local adaptation

When using the above estimated transfer matrix ***M5***, any change of geometry is perfectly controlled in a single step (Eq. 6) back to steady state. This principle of control is however not physiologic. Matrix ***M5*** contains many cross-terms where sensed signals in one wall (e.g. the LV wall) are used for feedback in another wall (e.g. an atrial wall). We assume that locally sensed signals can affect local structure only. So, no cross-terms are allowed between different walls. Furthermore, we assume that adaptation characteristics are the same for all walls since they all consist of comparable myocardial tissue. So, a new matrix ***M5*** *_u_* is defined, satisfying the latter conditions. In this matrix, all cross-terms between different walls are set to zero, leaving 5 similarly shaped sub-matrices along the diagonal, each of which corresponding to a particular wall. Using the condition that adaptation characteristics are the same for all walls, all sub-matrices are set identical to the universal matrix ***M*** according to Eq. (3).

The much smaller matrix ***M*** is designed to calculate local increments of 3 geometric parameters from the set of locally sensed variables. The matrix is found as the best fit matrix, using the method of Eq.(7), but now substituting vector lists of ***LnSens_m_*** and ln(***Geom_m_***) for all walls and all *m*. The latter vectors are 5 times shorter than vectors ***LnSens5*** *_m_* and ***Geom5*** *_m_*, respectively, while the list of vectors is 5 times longer. It is used:
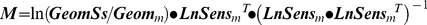
(8)Matrix ***M5_u_*** is composed of 5 copies of matrix ***M*** on the diagonal and zeroing all other elements. In simulating an adaptation step, from a certain geometry vector ***Geom5*** *_t_*, the new vector ***Geom5*** *_t_*_+1_ is found by replacement of matrix ***M5*** in Eq.(5) with ***M5*** *_u_*. Because ***M5_u_*** differs from ***M5***, vector ***Geom5*** *_t_*_+1_ will not be equal to the steady state vector ***Geom5Ss*** (Eq. 6). To analyze the way that adaptation approaches steady state, in a linearized form we write an adaptation step for vector ***Geom5*** as a multiplication with matrix ***H5***:
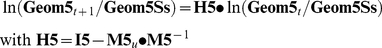
(9)Symbol ***I5*** represents the 15×15 identity matrix. In Eq. (9) vector divisions are component by component. Principles of control systems theory require all Eigen values of the 15×15 square matrix ***H5*** to be less than 1. If the latter condition is not satisfied, the related Eigen vector of ***Geom5*** *_t_* will diverge from the target vector ***Geom5Ss*** step by step by a factor equal to that Eigen value, implying instability of control by adaptation. We also have used a more qualitative approach to judge quality of control by checking that any deviation of geometry from the stable end solution should highly correlate with the calculated compensation.

Besides feedback coefficients, for the candidate sensed variables, target values (***SensRef*** in Eq. 2) have to be determined. Target values are chosen so that cardiac geometry is physiological in the steady state of adaptation. Whenever possible, for atrial and ventricular tissue the same target values are used. If however calculated steady state geometry of the atria appears non-physiologic, while ventricular geometry is physiologic, target values of the atria are adjusted so that atrial geometry becomes physiologic too. Matrix ***M*** *_u_* will however be the same for atria and ventricles under all circumstances.

## Results

Because the heart must withstand increased levels of hemodynamic load, a state of moderate exercise was assumed for simulation of adaptation. In Table 2, the stable reference state of geometric parameters of the five myocardial walls is presented together with some hemodynamic parameters. MyoM reference area indicates mid-wall area at a sarcomere length of 2.3 µm. ECM reference area indicates maximum mid-wall area during the cardiac cycle with exercise. Though adaptation is simulated with exercise, hemodynamic data are also presented for the state of rest. In the ventricular walls ECM size is about equal to MyoM size, whereas in the atria, ECM size is considerably larger than MyoM size. The latter finding is in agreement with earlier statements in the introduction.

ECM size is mostly determined by the state of maximum stretch which occurs at the beginning of ventricular diastole, when the atria are not active. During the ventricular fast filling phase atrial volumes decrease. Thereafter, at the beginning of atrial contraction, atrial volumes are lower than at the end of systole. Since MyoM size is mostly determined by the phase of contraction, MyoM area is lower than ECM area.

**Figure 4:**
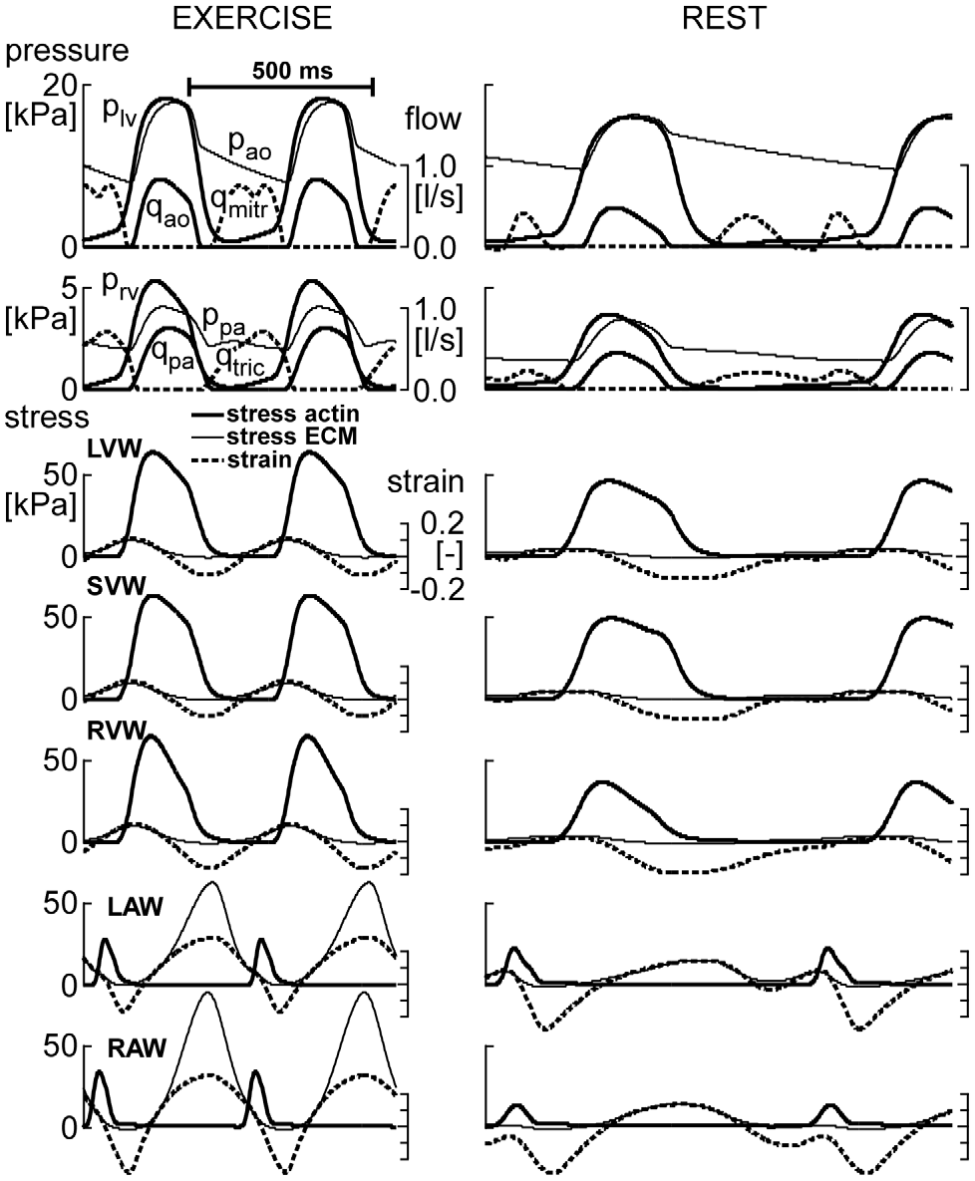
Simulation of circulation dynamics and cardiomechanics. With the CircAdapt model, beat-to-beat circulation dynamics are simulated at moderate exercise (left) and at rest (right). Top: Pressures in left/right ventricle (p_lv_/p_rv_) and aorta/pulmonary artery (p_ao_/p_pa_), and flows through aortic/pulmonary (q_ao_/q_pa_) and mitral/tricuspid valves (q_mitr_/q_tric_). Lower panels: Stresses in actin and ECM of the 5 cardiac walls and related fiber strains. For exercise and rest, amplitude and time calibrations are the same. Abbreviations LAW, RAW, LVW, SVW, RVW are defined in Fig. 2.

**Figure 5:**
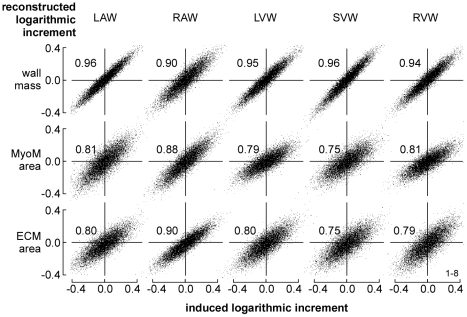
Reconstruction of cardiac wall geometry by mechano-sensing. A large number (10,000) of random increments of structural parameters was induced in all cardiac walls. In each wall, information about mechanical load of the tissue was obtained from 8 locally sensed variables (Table 2). With this information, best estimates were made of the originally induced increments. For the 3 structural parameters (vertical) and 5 walls (horizontal) reconstructed increment is plotted as a function of original increment. Numbers in the graphs indicate correlation coefficients.

In Fig. 4, simulated time courses of LV and RV hemodynamics are shown for moderate exercise and for the resting state. Besides, for all 5 cardiac walls, stress in the actin filaments of the myocytes (*S_act_*) and in the ECM (*S_ecm_*) are shown as a function of time. From the time courses expressing mechanical load, for each wall the set of 8 sensed variables (Table 2) was calculated for 10,000 random fractional increments in tissue volume, reference wall area of the MyoM and that of the ECM, respectively representing hypertrophy, dilation of the MyoM and dilation of the ECM. In Fig. 5, all available locally sensed variables were used to reconstruct the latter fractional increments. Reconstructed increments are plotted as a function of the true increments of these parameters for all myocardial walls. Most correlations are high. Correlations for mass of the RA wall (RAW) and area of the septal wall (SVW) are somewhat lower. Note also that various slopes differ from the line of identity.

In Table 3, the quality of control was quantified for the best performing combinations of sensed variables. Column 1 represents the minimum correlation coefficient *cor_min_* among the 15 graphs, examples of which are shown in Fig. 5. Column 2 represents maximum Eigen value *EV_max_* of matrix ***H*** (Eq. 9), characterizing convergence to steady state by closed loop control. In column 3 *svd3* represents the third Eigen value of matrix ***M_u_*** (Eq. 8) normalized to the sum of Eigen values. If this Eigen value equals zero, the matrix is singular, implying that corrections by control cannot be complete. So, we used *svd3* as a measure of the distance to matrix singularity.

**Table 3:** Quality of reconstruction with combinations of sensed variables.

*cor_min_*	*EV_max_*	*svd3*	*ID*	*S_ecm_*	*S_act_*	*S_int_*	*S_Ztit_*	*L_s,act_*	*L_s,int_*	*e_act_*	*w_stroke_*
		%		1	2	3	4	5	6	7	8
*3 sensed variables*											
0.71	1.11	10	**134**	x		x	x				
…											
0.62	0.99	13	**126**	x	x				x		
0.62	1.00	14	124	x	x		x				
0.60	1.01	11	125	x	x			x			
…											
*4 sensed variables*											
0.75	0.99	19	**1236**	x	x	x			x		
0.74	0.97	13	**1346**	x		x	x		x		
0.74	0.99	21	1234	x	x	x	x				
0.72	0.99	17	1235	x	x	x		x			
…											
*8 sensed variables*											
0.74	0.95	14	1–8	x	x	x	x	x	x	x	x

*cor_min_* = minimum correlation coefficient.

*EV_max_* = maximum Eigen value of feedback loop.

*svd3* = distance to matrix singularity.

*ID* = evaluated combinations identified by sensor numbers.

*S_ecm_*, *S_act_*, *S_int_*, *S_Ztit_*, *L_s,act_*, *L_s,int_*, *e_act_*, *w_stroke_* are sensed variables (Table 2).

When using a minimum of 3 sensed variables, the combination 134, i.e., *S_ecm,max_*, *S_int_* and *S_Ztit_*, rendered best correlation, but *EV_max_* exceeded unity. For combination 126, i.e., *S_ecm,max_*, *S_act,max_* and *L_s,int_*, correlation was somewhat lower, but *EV_max_* was lower than 1. Combinations 125 and 124 were nearly as good, showing that sensed signals *S_Ztit_*, *L_s,act_* and *L_s,int_* contributed similarly to the quality of control. When using 4 sensed variables, the combination 1236 rendered best correlation. With combination 1346 correlation was slightly lower, but *EV_max_* was better. Adding redundancy by using a 4th sensed variable elevated correlation, lowered *EV_max_* and increased *svd3*, thus indicating a clear improvement of control by adaptation. By using 8 variables, correlation did not improve much further. Strikingly, signal *S_ecm,max_* was needed in all well performing combinations, while *S_act,max_* was also often used. The signals systolic strain *e_act_* and stroke work density *w_stroke_* were of minor importance.

In Table 4 coefficients of transfer matrix ***M_u_*** are shown for the combinations 134, 126, 1236, and 1346, respectively (printed bold in Table 3). In all combinations, the effect of ECM-stress *S_ecm,max_* was found to increase tissue mass (hypertrophy) and dilate both MyoM and ECM. Increase of working length *L_s,int_* of the sarcomere, weighted with integrin stress (126, 1236, 1346), dilated MyoM so that sarcomere length returned to normal. Increase of active stress (126, 1236) increased wall mass and shrank both MyoM and ECM. Integrin stress *S_int_* (134, 1236, 1346) shrank the MyoM. Internal stress *S_Ztit_* in the Z-disk (134, 1346) increased wall mass and shrank the ECM. On average, coefficients related to *L_s,int_* are relatively high, indicating that this length was controlled in a relatively small range. In the right columns of Table 4, target values of the applied candidate variables are shown.

**Table 4:** Transfer coefficients from sensed variable to structural parameter.

*structural parameters→*	Δln(wall	Δln(MyoM	Δln(ECM	reference values		
	mass)	area)	area)	atria	ventr	unit
**transfer matrix ** ***M_u_*** ** (134)**	*not stable*					
Δln(*S_ecm,max_*)	+0.17	+0.07	+0.11	63	4.3	kPa
Δln(*S_int_*)	−0.10	−0.19	+0.00	4.8	4.8	kPa
Δln(*S_Ztit_*)	+0.12	+0.18	−0.12	1.8	3.6	kPa
**transfer matrix ** ***M_u_*** ** (126)**						
Δln(*S_ecm,max_*)	+0.03	−0.04	+0.14	63	4.3	kPa
Δln(*S_act,max_*)	+0.19	−0.17	−0.11	30	60	kPa
Δln(*L_s,int_*)	−0.31	+1.34	−0.40	2.1	2.1	µm
**transfer matrix ** ***M_u_*** ** (1236)**						
Δln(*S_ecm,max_*)	+0.19	+0.13	+0.09	63	4.3	kPa
Δln(*S_act,max_*)	+0.26	−0.10	−0.14	30	60	kPa
Δln(*S_int_*)	−0.20	−0.21	+0.06	4.8	4.8	kPa
Δln(*L_s,int_*)	+0.18	+1.84	−0.53	2.1	2.1	µm
**transfer matrix ** ***M_u_*** ** (1346)**	*best*					
Δln(*S_ecm,max_*)	+0.03	+0.22	+0.22	63	4.3	kPa
Δln(*S_int_*)	+0.02	−0.31	−0.09	4.8	4.8	kPa
Δln(*S_Ztit_*)	+0.74	−0.40	−0.57	1.8	3.6	kPa
Δln(*L_s,int_*)	−5.07	+4.83	+3.67	2.1	2.1	µm

**Figure 6:**
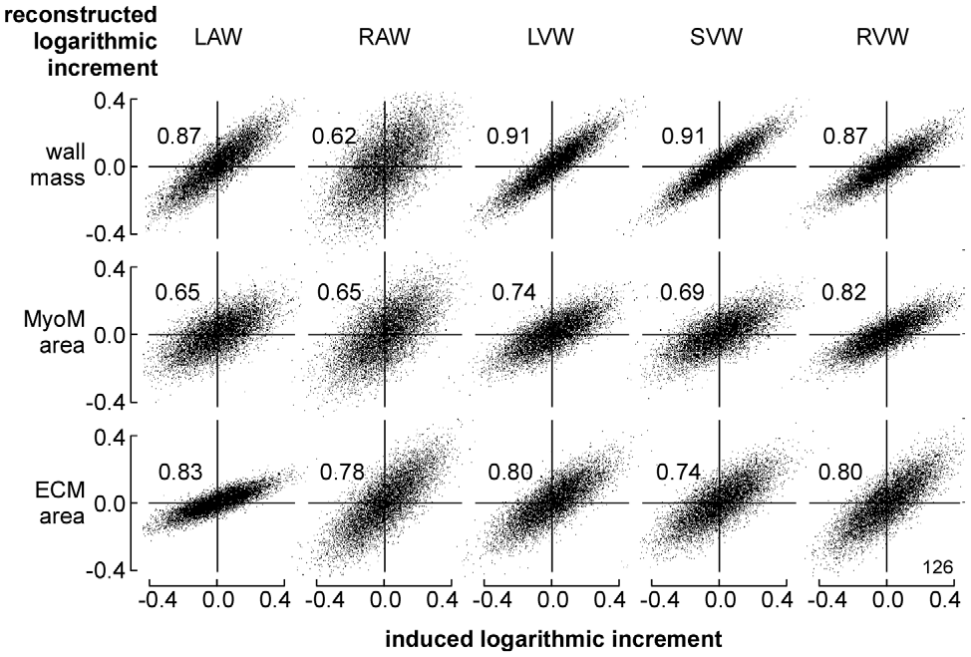
Reconstruction of wall geometry from 3 sensed variables (126). Mechano-sensed variables, used for feedback, are *S_ecm,max_*, *S_act,max_*, and *L_s,int_* according to Table 2. For explanation, see also Fig. 5.

In Fig. 6, reconstructed fractional increments of the structural parameters wall volume, MyoM area and ECM area are plotted as a function of the true increments, following the format of Fig. 5. Here, however, instead of using all available information the reconstruction was based on combination 126 with only 3 sensed variables, being ECM stress *S_ecm,max_*, active stress *S_act,max_*, and integrin stress weighted sarcomere length *L_s,int_*. Compared to Fig. 5, in Fig. 6 correlations were less, and the slopes varied more.

**Figure 7:**
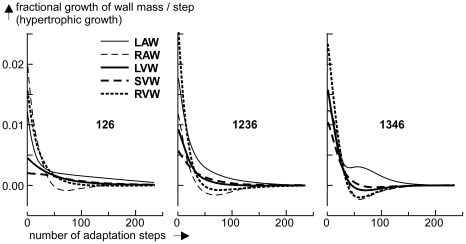
Simulation of hypertrophic growth of the five myocardial walls. The fractional change of wall mass per adaptation step is plotted as a function of the number of steps after 20% increase of cardiac output. The numbers 126, 1346 and 1236 refer to the applied combination of sensed variables, used for mechano-feedback (Table 3). After fast mass growth during the first 25 cycles, for combination 126 a slow component remains active for a long time. Convergence with 1236 is best. Convergence with 1346 is also fast, but there is more overshoot.

For selected combinations of sensed variables (134, 126, 1346, 1236), control of wall mass was simulated by closing the control loop, as indicated in Fig. 3. Next, cardiac output was increased by 20%, followed by carrying out 240 adaptation steps. In Fig. 7 fractional increments in tissue mass are shown as a function of the number of adaptation steps for all 5 myocardial walls. Since adaptation occurs on a much larger time scale than hemodynamic variations, in all simulations control of geometry was slowed down by multiplication of the calculated feedback correction (Eq. 3, 5) with a factor of 0.1.

Adaptation with combination 134 is not shown, because it did not converge due to the high value of *EV_max_*. With combination 126, control was stable, but there was a component with slow convergence, clearly shown by left atrial wall mass. After 240 control steps, steady state was still not reached. By adding feedback with *S_int_* (1236), convergence of control was much faster, as shown by convergence of all wall masses already after 180 steps. After replacing feedback of *S_act_* with that of *S_Ztit_* (1346) convergence was slightly faster, but there was more overshoot early in the response after about 40 steps. Apparently, when using 4 instead of 3 feedback factors, convergence was faster. Corresponding data on adaptation of MyoM and ECM area are not depicted, but convergence behavior was similar for those variables.

Sensitivity to changes in target variables has been investigated by variation of the target values of combination 1236, i.e., *S_ecm,max_*, *S_act,max_*, *S_int_* by 20%, and *L_s,int_* by 5%. In the simulations, relative changes in wall volume, MyoM area and ECM area were recorded. In Table 5, data are presented as a sensitivity matrix of ratio of logarithmic changes. Values of 1.0, 2.0 and −1.0 represent linear, quadratic and reciprocal dependency, respectively. Apparently, geometric parameters are sensitive to changes in sarcomere length. Increase of the target value of working sarcomere length implies decrease of needed mass (except for the RAW) and MyoM area, while ECM area also decreases, albeit somewhat less. Most magnitudes of dependency exponents are smaller than 1, implying that these dependencies are moderate and non-critical. Increase of ECM stress implies general shrinkage of the heart. Decrease of active stress (*S_act,max_*) causes wall mass to increase and cavities to decrease in size, i.e., known as concentric hypertrophy. The effect of integrin stress is about opposite to that of ECM stress.

**Table 5:** Sensitivity of steady state geometry to changes in target values.

Exponents of dependency					
*resulting changes in:*		*varied target values:*			
*wall*	*variable*	*S_ecm,max_*	*S_act,max_*	*S_int_*	*L_s,int_*
LAW	*V_wall_*	−1.81	−1.53	0.70	−0.68
	*A_myom_*	−1.47	0.20	0.61	−3.57
	*A_ecm_*	−1.44	0.20	0.29	−1.01
RAW	*V_wall_*	0.23	−1.54	0.21	3.23
	*A_myom_*	−0.38	0.21	0.42	−3.58
	*A_ecm_*	−0.36	0.14	0.17	−1.10
LVW	*V_wall_*	−0.83	−0.60	1.18	−4.23
	*A_myom_*	−0.39	0.29	0.69	−4.19
	*A_ecm_*	−0.38	0.34	0.43	−1.88
SVW	*V_wall_*	−1.18	−0.64	1.25	−4.96
	*A_myom_*	−0.67	0.15	0.74	−4.53
	*A_ecm_*	−0.65	0.20	0.48	−2.23
RVW	*V_wall_*	−0.24	−0.82	0.97	−2.64
	*A_myom_*	−0.34	0.26	0.65	−4.14
	*A_ecm_*	−0.34	0.30	0.40	−1.87

LAW, RAW, LVW, SVW, RVW = myocardial walls.

*V_wall_* = wall volume, *A_myom_* = MyoM area, *A_ecm_* = ECM area.

**Figure 8:**
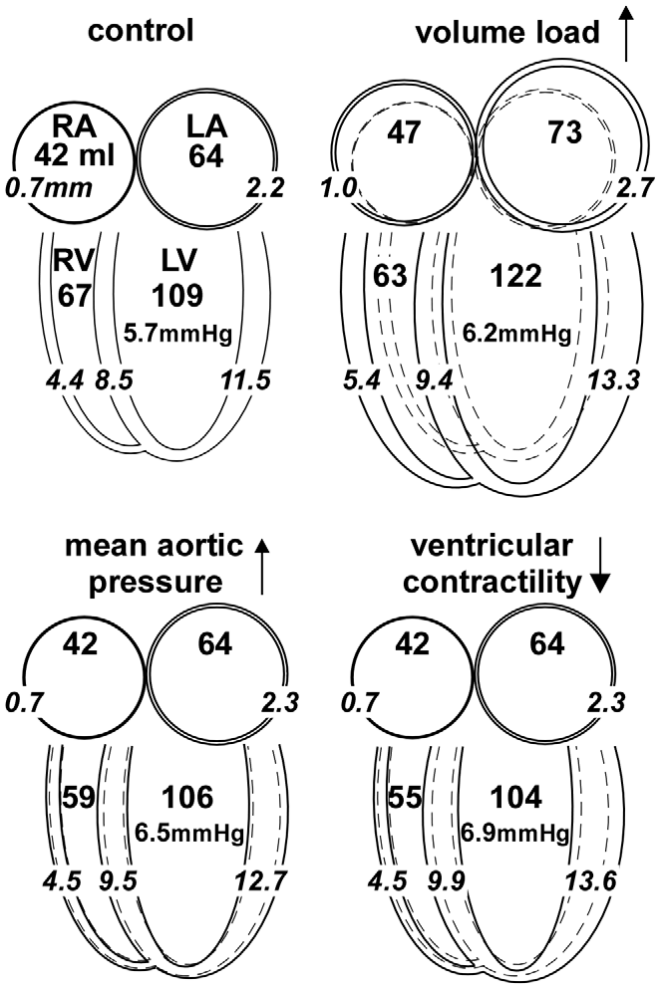
Adaptation to a 20% change of pump load and contractility. Mean volumes of right and left atria (RA, LA) and right and left ventricles (RV, LV), respectively, are indicated by the larger numbers in ml. Mean thicknesses of atrial and ventricular walls are indicated by italic numbers in mm. Drawn changes in geometry are exaggerated for better visibility. End-diastolic pressures are indicated in mmHg. With 20% increase of stroke volume, all volumes and thicknesses increase (eccentric hypertrophy), except for RV volume. With 20% increase of mean aortic pressure, walls thicken, while geometry does not change much. With a 20% decrease of LV and septal contractility, these walls thicken, while further geometry is preserved.

Finally, we simulated adaptation for several clinically relevant phenomena, i.e. chronic volume load, hypertension and decreased contractility of the left ventricle (LV). In Fig. 8, wall volumes and thicknesses are averaged over the cardiac cycle, and indicated by numbers in ml and mm, respectively. Furthermore, LV end-diastolic pressure is indicated in mmHg. With a 20% increase of volume load, cavity volumes increase and walls thicken, while end-diastolic pressure slightly rises. These changes represent eccentric hypertrophy. With a 20% increase of mean aortic pressure, wall thicknesses of LVW and SVW increase, while other changes in geometry are minor. LV end-diastolic pressure rises. These changes represent concentric hypertrophy. With a decrease of contractility, changes in geometry are similar to those of concentric hypertrophy. LV end-diastolic pressure rises further, indicating loss of pump function. Note that in these cases, geometry is fully adapted. With pathology, adaptation is often incomplete, resulting in more severe signs of function decrease.

## Discussion

Adaptation of cardiac geometry to mechanical load is considered a homeostatic system. In the CircAdapt model of cardiovascular mechanics and hemodynamics, geometry of left and right atrial wall and left, right and septal wall of the ventricles were used to simulate pressures and volumes in heart and blood vessels as a function of time. In atrial and ventricular walls, a group of 8 mechano-sensed signals was calculated. A matrix of transfer coefficients simulated the network of mechano-transductive pathways, leading to structural changes, i.e., changes in wall mass, in area of the myocyte matrix (MyoM) and in area of the extra-cellular matrix (ECM). The control loop was closed by using the calculated structural changes to determine a new geometry for all cardiac walls.

Various combinations of mechano-sensed signals were evaluated on quality of control. One combination of 3 sensed signals was found, resulting in high stability of control of geometry for all cardiac walls (Fig. 5, 7), using the criterion that the highest Eigen value of matrix ***H*** (Eq. 9) should be smaller than one. By adding a 4^th^ signal, several combinations were found to satisfy the latter condition (Table 3, Fig. 5). With four signals, control appeared faster and more accurate; showing that introduction of redundancy in feedback improves control properties.

In our search, the combination 1236 (Table 3, Fig. 5, 7) appeared best. Sensed signals were maximum ECM stress (*S_ecm,max_*), maximum active stress (*S_act,max_*), integrin stress (*S_int_*) between ECM and MyoM and integrin stress weighted sarcomere length (*L_s,int_*). With this combination, *S_ecm,max_* is predicted to induce hypertrophy and dilate both MyoM and ECM. The latter finding is in agreement with that in a longitudinal study (12 weeks) by Donker et al. [29] on dogs, subjected to volume overload after reduction of heart rate by atrio-ventricular conduction blockade. The rate of increase of LV mass appeared synchronous and proportional with the level of end-diastolic stress, which finding agrees also with the finding that stretch of myocardial cells decelerates breakdown of contractile proteins [30]. In the Donker et al. study, end-diastolic volume increased gradually while end-diastolic stress decreased, thus indicating that the ECM dilated just as predicted. During the phase of LV dilation, peak-systolic stress did not change, suggesting that the MyoM dilated with increase of end-diastolic volume. However, complete agreement could not be proven because information about integrin stress and sarcomere length was not reported. Dilation of ECM after volume load has been reported in other studies [17], [31] as well. We predicted that peak systolic stress *S_act,max_* should also induce hypertrophy and shrinkage of the MyoM and ECM. In patients, pressure overload is found to induce systolic stress overload, followed by concentric hypertrophy [32], [33], just as found in our simulation (Table 5). Furthermore, we predicted that *S_int_* should shrink both wall mass and MyoM area, and that sarcomere stretch, as indicated by a long *L_s,int_*, should induce hypertrophy, dilation of MyoM and shrinkage of ECM. About the effect of integrin stress (*S_int_*) little is known quantitatively, because this stress cannot be measured directly.

Several combinations of sensed variables were found with stable control of geometry in all walls (Table 3). In nearly all well-performing combinations sensing stress in the ECM and MyoM (*S_ecm,max_*, *S_act,max_*) appears important. Necessary information about sarcomere length may be obtained from one variable out of the group *S_Ztit_*, *L_s,act_* and *L_s,int_*. Information about stress *S_int_* between MyoM and ECM, which is likely sensed by integrins, appeared to improve stability of control. Information on active stress weighed strain (e*_act_*) and stroke work (*w_stroke_*) seemed of minor importance.

As mentioned in the introduction, in the real heart, more than three sensing mechanisms are reported to exist, indicating substantial redundancy in mechano-sensing. Redundancy improves robustness of control by use of alternative pathways if one of the primary pathways is blocked. The advantage of robustness is shown by comparing case 1236 and 126 in Fig. 7. By missing of sensed signal *S_int_* the system remains stable, albeit performance of the system is not as good, as shown by a substantially slower component in corrective control.

Numerous reports [1], [3], [11], [22] have been presented about the intricate network of many different and partly overlapping chemical pathways, converting sensed information to structural change. We think that redundancy of the network renders so many degrees of freedom in design that various network solutions satisfy the condition of stable control of geometry. Thinking in terms of the evolution of species, it is to be expected that survival rate improves by proper control of cardiac geometry. So, in different species, the network of chemical pathways and mutual connections may be different with the restriction that the condition of stable control is satisfied.

In the model, adaptation may interfere with beat-to-beat changes of hemodynamics. In the real situation, adaptation is on a much slower time scale than cardiac beat-to-beat dynamics. Instead of using the best estimate of the steady state in a single, fast control step, the speed of step by step control was diminished by multiplication with a factor 0.1. Thus, adaptation effects became slow relative to the beat to beat changes of hemodynamics in order to avoid unphysiological dynamic interference. After all, real adaptation is very slow as compared to beat to beat changes. Further reduction of the speed of adaptation did not affect the simulated result anymore. Comparing the speed of control for case 1236 in Fig. 7 with measurements by Donker et al. [29], we deduced that 3 controls steps are about equivalent with 1 day of adaptation.

In Fig. 8, changes in geometry are shown after adaptation to changes in load. Wall volumes and thicknesses are averaged over the cardiac cycle, and indicated by numbers in ml and mm, respectively. Furthermore, LV end-diastolic pressure is indicated in mmHg. With a 20% increase of volume load, cavity volumes increase and walls thicken, while end-diastolic pressure slightly rises. These changes represent eccentric hypertrophy. With a 20% increase of mean aortic pressure, wall thicknesses of LVW and SVW increase, while other changes in geometry are minor. LV end-diastolic pressure rises somewhat. These changes represent concentric hypertrophy. With a decrease of contractility, changes in geometry are similar to those of concentric hypertrophy. LV end-diastolic pressure further rises, indicating loss of pump function. Note that in these cases, geometry is fully adapted. With pathology, adaptation is often incomplete, resulting in more severe signs of function decrease.

Pathologic decrease of contractility of the myocardial tissue rendered concentric hypertrophy (Table 5). Apparently, more myocardial mass was needed to satisfy the needs for pumping. A side effect of the increase of wall thickness is stiffening of the wall, thus hampering diastolic filling. Clinically, the resulting elevation of diastolic pressure is often interpreted as a sign of maladaptation. When simulating a further decrease of contractility, the LV appeared not able to contract sufficiently, leading to ECM dilatation. The latter state is clinically interpreted as dilated cardiomyopathy.

In summary, we succeeded in simulating adaptation of whole heart geometry to mechanical load, using a computer model. A system of local mechano-feedback is found that is universal to atrial and ventricular myocardium, resulting in stable control of geometry. Mechano-sensing by the myocardial cells induces local changes in tissue mass (hypertrophy), myocyte matrix (MyoM) extent and extra cellular matrix (ECM) extent. Macroscopically, these changes indicate control of wall mass, wall area of the MyoM and wall area of the ECM. At least three mechanical variables should be sensed for proper control of geometry. Redundancy, introduced by additional variables, improves accuracy and reliability of control. A best-fit matrix of coefficients, quantifying transfer of sensed signals to structural change, was estimated by computer simulation. In a search to find best control properties with the use of just four sensed variables, we found a solution that closely resembles physiology of adaptation. With this solution, sensed maximum ECM stress induces hypertrophy and dilates both MyoM and ECM. Sensed maximum myocyte stress induces hypertrophy also, but shrinks MyoM and ECM. Sensed stress in the integrins, bridging the ECM to the MyoM, shrinks both wall mass and MyoM. Elongation of sarcomeres induces increase of wall mass, MyoM dilation and ECM shrinkage. Different combinations of mechano-sensors were found that satisfied the condition of stable control of geometry. Thus, we expect that for the various species evolution may have selected different solutions of mechano-adaptation.

## Supporting Information

Text S1 CircAdaptManual.doc is a short manual of the CircAdapt model, as programmed in MATLAB.
(DOC)

Text S2 CircAdapt.txt contains the source text of all MATLAB m-files sequentially with file separating markers.
(TXT)

Text S3 UnpackCircAdapt.txt is a text file, to be converted to a MATLAB m-file by changing the ‘.txt’ ending part to an ‘.m’ ending part. The file is needed to unpack all program files of the CircAdapt model from the file CircAdapt.txt. The unpacking procedure is described in more detail in the CircAdapt manual.
(TXT)
