# Supplementary material for: Control of Whole Heart Geometry by Intramyocardial Mechano-Feedback: A Model Study
Source: PLoS Comput Biol. 2012 Feb 9;8(2):e1002369. doi: 10.1371/journal.pcbi.1002369 (PMC3276542; doi:10.1371/journal.pcbi.1002369)
Supplement: Text S1 — CircAdaptManual.doc is a short manual of the CircAdapt model, as programmed in MATLAB. (DOC) [file pcbi.1002369.s001.doc]

# The CircAdapt model

T. Arts, T. Delhaas, F. Prinzen, J. Lumens

Maastricht University, The Netherlands

The CircAdapt model is a lumped model of heart and circulation, simulating beat-to-beat hemodynamics and mechanics. The basis of the model has been presented earlier (*Arts T, Delhaas T, Bovendeerd P, Verbeek X and Prinzen FW. Adaptation to mechanical load determines shape and properties of heart and circulation, the CircAdapt model. Am J Physiol Heart Circ Physiol 288: 1943-1954, 2005; Lumens J, Delhaas T, Kirn B, Arts T (2009) Three-wall segment (TriSeg) model describing mechanics and hemodynamics of ventricular interaction. Ann Biomed Eng 37: 2234-2255*). The model is composed of a few types of elements, i.e. muscular chambers, elastic tubes, valves and peripheral resistances. Chambers are composed of curved sheets. The size of the constituting elements adapts to mechanical load, as generated by the model itself.

## Unpack CircAdapt

In presenting CircAdapt, references are made to the program source file. The program is written and tested in MATLAB version R2007b with Windows 7 operating system. For installing the program the following files are available:

- CircAdapt.txt
- UnpackCircAdapt.txt

CircAdapt.txt contains the text of all needed *.m-files, terminated by separator codes. The filename UnpackCircAdapt.txt should be changed to UnpackCircAdapt.m. Then it is a short MATLAB-program file, suited to unpack CircAdapt.txt.

The following steps are to be made (Windows environment):

- Place CircAdapt.txt and UnpackCircAdapt.m in an empty new folder.
- Start up MATLAB with the folder as working directory. Execute command
  >> UnpackCircAdapt <Enter>
- All m-files are placed in a new folder CircAdapt in this folder.
- Change working directory by
  >> cd CircAdapt <Enter>
- Startup a first simulation with CircAdapt by
  >> CircAdaptMain <Enter>
- 2 more times <Enter>
- Two initial beats are simulated. Some results are shown in Figure 1.

CircAdapt installation is ready.

## Setup of the CircAdapt model

The model is schematically presented in Fig. (1).


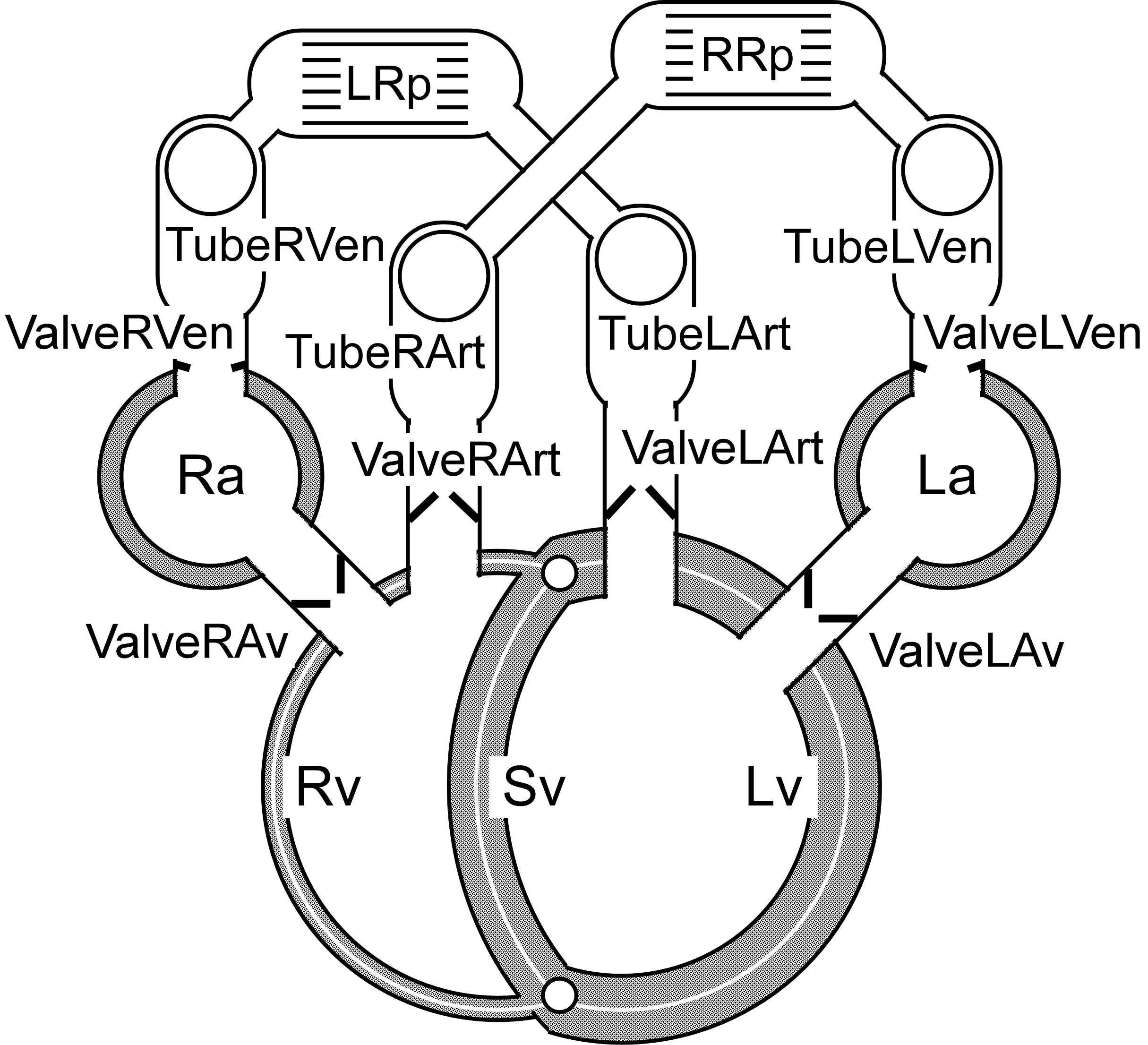


Fig 1: Schematic presentation of the CircAdapt model of the whole circulation. Meaning of the symbols: Rv, Lv and Sv, = right and left ventricular cavities and free walls, and septal wall, respectively; Ra, La = right and left atrium; ValveRVen, ValveRAv, ValveRArt = Ra inflow channel, tricuspid valve and pulmonary valve; ValveLVen, ValveLAv, ValveLArt = La inflow channel, mitral valve and aortic valve; TubeRVen, TubeRArt, TubeLVen, TubeLArt = systemic veins, pulmonary artery, pulmonary veins and aorta; RRp, LRp = pulmonary and systemic peripheral resistance.

The core of the model is a system of differential equations with a limited number (e.g. 30) of state variables (SVar). The set of state variables uniquely defines the state of the whole circulation. The set of time derivatives of the state variables is a unique function of the set of state variables, represented by the program part ‘SVarDot’. The system of differential equations has been solved in Matlab (function ‘ode113’) by scaling of the state variables to values around 1. For scaling program files Par2SVar, Par2SVarDot and SVar2Par are used. All parameters and variables are stored in a comprehensive data structure ‘Par’.

The Rv and Lv cavity are enclosed by three walls, i.e. Rv free wall, septal wall (Sv) and Lv free wall. The La and Ra cavities are enclosed by single walls La and Ra, respectively. Walls are modeled as sheets. Sheet area contracts if the myofibers in the sheets contract. Entrances of valves and tubes are dealt with as non-contractile sheets, encapsulating the cavities together with the contractile sheets. Stress in the myofibers depends on strain and strain rate according to physiological experiments on isolated myofibers.

As seen from the heart, arterial inlets and venous outlets are represented as Tube-elements. Tubes are three-element impedances, consisting of a resistive characteristic impedance in series with a compliance and a peripheral resistance. The peripheral resistance is a separate element, connecting the arterial vessel bed to the venous vessel bed. Venous inlets to the atria are represented similar to arterial outlets with the only difference the direction of average flow. So, changing pressures in the atria induce flow waves back into the veins.

Like in the real circulation, regulation of arterial blood pressure requires the possibility to vary circulating blood volume. In the model, blood pressure is regulated by adding blood volume to the venous compartments.

## List of symbols and abbreviations

Elements and variables have been named carefully.

### Elements

An element is named as a concatenation of terms, each having the first letter capitalized. The structure is generally as follows:

<Element type> <Left/Right> <Location>

The following abbreviations for Element types have been used:

- Valve
- Tube

The element type of myocardial cavity or wall has not been named.

The following abbreviations for left/right have been used:

- L: left
- R: right
- S: septal

The following abbreviations for anatomical location have been used:

- Art: arterial
- Ven: venous
- a: atrium
- v: ventricle
- Av: atrial-ventricular
- ASD : atrial-septal defect
- VSD : ventricular-septal defect
- DUCT: ductus arteriosis
- Peri: pericardium
- Sarc: sarcomere/sheet
- Rp: peripheral resistance
- m: mid-wall of sheet

### Variables

Variables are named, starting with their physical symbols, followed by further specifications. All variables are expressed in SI-units:

| ***Symbol*** | ***Unit*** | ***Description*** |
| --- | --- | --- |
| X, Y, Len | m | Length |
| A | m^2^ | Area |
| V | m^3^ | Volume |
| C | m^-1^ | Curvature= 1/radius |
| C | - | Time-variant contractility of sarcomere |
| p | Pa= Nm^-2^ | Pressure |
| q | m^3^s^-1^ | Flow |
| R | Pa m^-3^s | Hemodynamic resistance |
| Z | Pa m^-3^s | Hemodynamic impedance |
| L | Pa m^-3^s^2^ | Inertia |
| t, Tau, T | s | Time |
| T | Nm^-1^ | Wall tension |
| rhob | kg m^-3^ | Density of blood |
| Ls | micrometer | Sarcomere length (not SI!) |
| Ef | - | Strain of fibers |
| Sf | Pa= Nm^-2^ | Stress in fibers |
| v | ms^-1^ or micrometer/s | Velocity |
| *For handling of the differential equations:* | | |
| SVar | [-] | State variable (normalized by Par.Scale) |
| <var>Dot | [-] | Time derivative of <var> |

Variables related to setpoints of adaptation are stored in ‘xxx.Adapt’ records.

## The program

Properties of all elements are stored in tree-organized structure Par, globally accessible throughout most of the program and functions.

Par =

t: [851x1 double] time, 851 samples in two heart beats

tDot: 1 dt/dt

ValveLVen: [1x1 struct] ValveLeftVenous= inlet LA

ValveLAv: [1x1 struct] ValveLeftAtrioVentricular= mitral

ValveLArt: [1x1 struct] ValveLaftArterial= aortic

ValveRVen: [1x1 struct] ValveRightVenous= inlet RA

ValveRAv: [1x1 struct] ValveRightAtrioVentricular= tricuspid

ValveRArt: [1x1 struct] ValveRightArterial= pulmonary

ValveASD: [1x1 struct] ValveAtrialSeptalDefect

ValveVSD: [1x1 struct] ValveVentricularSeptalDefect

ValveDUCT: [1x1 struct] ValveDuctusArterios

TubeLArt: [1x1 struct] TubeLeftArterial= aorta

TubeLVen: [1x1 struct] TubeLeftVenous= pulmonary veins

TubeRArt: [1x1 struct] TubeRightArterial= pulmonary artery

TubeRVen: [1x1 struct] TubeRightVenous= systemic veins

LRp: [1x1 struct] LeftPeripheralResistance

RRp: [1x1 struct] RightPeripheralResistance

La: [1x1 struct] Left atrium (cavity+wall)

Ra: [1x1 struct] Right atrium (cavity+wall)

Lv: [1x1 struct] Left ventricle (cavity+wall)

Rv: [1x1 struct] Right ventricle (cavity+wall)

Sv: [1x1 struct] Ventricular septum (wall only)

Peri: [1x1 struct] Pericardium

General: [1x1 struct] General information

Scale: [30x1 double] Scaling factors from physics<->DiffEq

SVar: [851x30double] State Variables (30)

SVarDot: [851x30double] d/dt(State Variables)

Adapt: [1x1 struct] Auxilary record for actions of adaptation

### Examples of data, stored in elements

Inlet orifice of pulmonary veins into left atrium:

Par.ValveLVen

rhob: 1050 Density of blood (SI-units)

q: [851x1 double] Flow trough valve (SVar)

qDot: [851x1 double] d/dt Flow

Prox: 'TubeLVen' Proximal element

Dist: 'La' Distal element

AOpen: [851x1 double] Area forward flow orifice

ALeak: 5.0000e-004 Area backward flow orifice

Len: 0.0201 Length flow channel

L: [851x1 double] Inertia

Inlet of aorta:

Par.TubeLArt

k: 8 Stiffness exponent of wall material

Adapt: Substructure of adaptation parameters

- WallStress: 500000 Maximum wall stress

- vFlowMean: 0.1700 Mean flow velocity

- vImpact: 3 Shock wave velocity due to body movement

q0: 8.5000e-005 Mean flow at rest

rhob: 1050 Density of blood

p0: 1.2288e+004 Mean pressure at rest

Len: 0.4001 Effective length of vessel (compliance)

V: [851x1 double]Volume of lumen

VDot: [851x1 double]d/dt lumen volume

pMax: 3.2092e+004 Maximum pressure

AWall: 1.1890e-004 Cross-sectional area of wall

A0: 4.9483e-004 Mean cross-sectial area at rest

A: [851x1 double]Cross-sectional area

p: [851x1 double]Pressure of compliance

Z: [851x1 double]Impedance

pIn: [851x1 double]Pressure at entrance

qRemod: [851x1 double]Flow, used for diameter adaptation

Left ventricular cavity and Lv free wall together

Par.Lv

Sarc: [1x1 struct] Mechanical properties of sarcomere

AmRef: 0.0103 Reference of mid-wall surface area

AmDead: 0.0020 Non-contractile area (Valve openings)

VWall: 1.2398e-004 Wall volume

V: [851x1 double] Cavity volume

VDot: [851x1 double] d/dt Cavity volume

Vm: [851x1 double] Volume enclosed by midwall

Xm: [851x1 double] Central bulge distance of midwall surface

Ym: [851x1 double] Radius of midwall at the junctional edge

Am: [851x1 double] Mid-wall surface area of wall

Cm: [851x1 double] Curvatur=1/radius of midwall

T: [851x1 double] Wall tension [N/m]

pTrans: [851x1 double] Transmural pressure

DTDAm: [851x1 double] dTension/dArea compliance

p: [851x1 double] Pressure at central cavity

A: [851x1 double] Crude estimate of cavity cross-section

Z: 0 Source impedance

pIn: [851x1 double] Pressure at inlet and outlet

qRemod: [851x1 double] --- (see Tube)

Y: [] Reserved for Septum mechanics

YDot: [] Reserved for Septum mechanics

Tau: [] Reserved for Septum mechanics

nSarc: 1 Number of patches

EAmRef: 0 Reserved for Septum mechanics

Myofiber and Patches of the Lv wall

If Lv.nSarc>1 then data in columns refer to each patch

Par.Lv.Sarc

Adapt: [1x1 struct] Record of adaptation parameters

ActivationDelay: [3x1 double] Time of mechanical activation

Ef: [851x1 double] Natural myofiber strain

LsRef: 1.9973 Reference sarcomere length [um]

Ls0Pas: 1.8000 Zero passive stress sarcomere length

dLsPas: 0.6000 Passive stiffness non-linearity [-]

SfPas: 4.1975e+003 Passive stiffness factor [Pa]

Lsi0Act: 1.5100 Zero active stress sarcomere length

LenSeriesElement: 0.0400 Length series elastic element [um]

SfAct: 1.2000e+005 Maximum isometric active stress [Pa]

vMax: 7.0000 Maximum velocity of shortening [um/s]

TimeAct: 0.4250 Duration activation [s]

TR: 0.2500 Rise time factor [-]

TD: 0.2500 Decay time factor [-]

C: [851x1 double] Contractility [-]

CDot: [851x1 double] dC/dt [1/t]

CRest: 0.0200 Diastolic rest contractility [-]

Lsi: [851x1 double] Unloaded sarcomere length [um]

LsiDot: [851x1 double] dLsi/dt [um/s]

Ls: [851x1 double] Sarcomere length [um]

SfPasT: [851x1 double] Stress in passive matrix [Pa]

Sf: [851x1 double] Total myofiber Gauchy stress [Pa]

DSfDEf: [851x1 double] Sarcomere stiffness [Pa]

(Patch data: see Par.Lv for meaning)

Am: [851x1 double]

Cm: [851x1 double]

VWall: 1.2398e-004

AmRef: 0.0103

T: [851x1 double]

pTrans: [851x1 double]

DTDAm: [851x1 double] Patch stiffness dT/dAm

The program uses the Par-structure, stored in file ‘Par.mat’ in the working directory. During execution of the program, Par is globally accessible, allowing derivation of information everywhere in the program about any location in the circulation.

### Using the program

### Introduction

For a simple start do the following.

Store all program files (*.m-files) in a program directory (e.g. called ProgDir).

Start up MatLab.

Go to a working directory.

Open a path to the program directory by >>addpath ProgDir

If the file ParRef with the reference simulation is not present, this file has to be made (see below), else:

Start the program by typing:

>> CircAdaptMain <Enter>

[N]ew, [R]eference, [L]oad, [C]ontinue <Enter>: r

type: R (Reference) to load ParRef.mat, representing a standard state of the circulation.

[P]ressure (kPa): 12.2

[F]low (ml/s): 85

cycle [T]ime (ms): 850

[D]uration simulation (s): 1.275

Adapt n[O]ne [R]est,[E]xercise : Adapt0

Faster steady state [Y]/[N] : N

<Enter> = Continue

Choose Letter <Enter>:

If nothing needs to be changed (default), press <Enter>

t= 0; Time to go= 1.275

Flow RVen RArt LVen LArt Err (ml/s), ErrFlow :

84.82 84.95 84.88 84.88 0.0006292

Error Steady State [1e-4]= 1

t= 0.85; Time to go= 0.425

Flow RVen RArt LVen LArt Err (ml/s), ErrFlow :

85.02 85.05 85.06 85.06 0.0005232

Error Steady State [1e-4]= 1

Differential equation has been solved

After each simulated beat, mean flow through four cardiac valves are printed out to judge the presence of steady state. Moreover, hemodynamic variables are plotted for the just simulated beat. Time ‘t’ increases, while the ‘time to go’ decreases until the end of the simulation.

The model will simulate 2 beats (default). A selection of results is shown graphically in figure 1. All units are shown in SI-units. Calibration factors are shown at the top of the figure.

Upper left pane: PV-loops of Lv, Rv, La and Ra. Atrial pressures are multiplied by 10 for better visibility. Upper mid pane: stress(strain) plot of myofibers. Zero strain refers to sarcomere length 2um. Lower left pane: diastolic pressures in Lv, Rv, La, Ra, LVen, RVen and pericardium. Right pane: top tracings refer to left hemodynamics, i.e. pressures and volumes of Lv,La, aortic pressure, venous inflow, mitral flow and aortic flow. At lower left, data are similar, but now derived from the right side.

### Making the file ParRef.mat, containing the reference state

When starting with a new simulation without history, it is convenient to make the file ParRef.mat to define the steady state reference state. The new circulation should adapt to the resting condition in steady state by adaptation of blood vessel diameters to flow. Next in the exercise state with 3-fold flow and 2-fold heart rate, cardiac geometry and wall thickness of the blood vessels adapt to mechanical load. Next the file ParRef is made by

>> save ParRef Par **<Enter>**

This procedure can be carried out by hand. For convenience the script MakeParRef.m has been added. Here, CircAdapt is doing its adaptation work.

>> MakeParRef **<Enter>**

The whole adaptation procedure takes quite long (about 200 beats, 10-20 min simulation time) because steady state is needed for adaptation.

### Intervention by changing parameter setting

Any parameter in the circulation can be changed. After changing a parameter setting in the structure Par, this structure should be stored as file ‘Par.mat’, because each simulation starts from reading this file. Below an example has been shown to simulate aortic valve stenosis.

Execute a normal beat by <CircAdaptMain> and <Enter>’s. Now, structure ‘Par’ is in memory. Make a change to aortic valve stenosis by

>> Par.ValveLArt.AOpen=0.2*Par.ValveLArt.AOpen;

>> save Par Par

Increase simulation time to 10s as follows:

>> CircAdaptMain **<Enter>**

[N]ew, [R]eference, [L]oad, [C]ontinue <Enter>: **<Enter>**

[P]ressure (kPa): 12.2

[F]low (ml/s): 85

cycle [T]ime (ms): 850

[D]uration simulation (s): 1.275

Adapt n[O]ne [R]est,[E]xercise : Adapt0

Faster steady state [Y]/[N] : N

<Enter> = Continue

Choose Letter <Enter>: **d**

Duration of simulation (s): **10**

Similarly, starting time may be set to zero by:

>> Par.t(end)=0;

>> save Par Par

### Starting from New, and using adaptation

The reference file has been obtained by starting from scratch, followed by adaptation of the myocardium to mechanical load under hemodynamic exercise conditions. Next vessel diameters will be adapted to flow under resting conditions. This procedure is repeated a few times, leading to the reference ‘Par’-structure, which is stored as file ParRef.mat. Note that the scratch state at the beginning is not influencing the final result. However, the scratch state should be in a range that the solution is stable, allowing convergence to a stable physiologic state.

>> CircAdaptMain **<Enter>**

[N]ew, [R]eference, [L]oad, [C]ontinue <Enter>: **N**

Number of state variables: 30

Start with simulation from scratch by typing N. The number of state variables is shown. The command WriteSVarFiles is executed to write the script files for conversion of simulated variables to Par.SVar and Par.SVarDot, to be used to solve of the equations by standard MatLab solvers (ode113).

[P]ressure (kPa): 12.2

[F]low (ml/s): 85

[T]ime of beat (ms): 850

[D]uration simulation (s): 1.275

Adapt n[O]ne [R]est,[E]xercise : Adapt0

Faster steady state [Y]/[N] : N

<Enter> = Continue

Choose Letter <Enter>: **<Enter>**

t= 0; Time to go= 1.275

Adapt vascular properties by adaptation at rest:

>> CircAdaptMain **<Enter>**

...

Adapt n[O]ne [R]est,[E]xercise : Adapt0

Faster steady state [Y]/[N] : N

<Enter> = Continue

Choose PFTDRES<Enter>: **R**

Now the menu shows the change in adaptation property.

...

Adapt n[O]ne [R]est,[E]xercise : **AdaptRest**

Press <Enter>. Wait for many beats, while adaptation takes place.

Now bring to exercise by 3-fold increase of blood flow, 50% reduction of cycle time. Simulate with ‘Faster steady state’ option or set duration to 20 s.

>> CircAdaptMain <Enter>

[P]ressure (kPa): 12.2

[F]low (ml/s): **255**

cycle [T]ime (ms): **425**

[D]uration simulation (s): 1.275

Adapt n[O]ne [R]est,[E]xercise : Adapt0

Faster steady state [Y]/[N] : **Y**

<Enter> = Continue

Choose Letter <Enter>:

Adapt to Exercise condition:

>> CircAdaptMain

[N]ew, [R]eference, [L]oad, [C]ontinue <Enter>:

[P]ressure (kPa): 12.2

[F]low (ml/s): 255

cycle [T]ime (ms): 425

[D]uration simulation (s): **42.6**

Adapt n[O]ne [R]est,[E]xercise : **AdaptExc**

Faster steady state [Y]/[N] : N

<Enter> = Continue

Choose Letter <Enter>: **<Enter>**

After many beats, convergence occurs (ErrSheet<0.001). If convergence is not yet complete, repeat an adaptation session to exercise.

Bring back to rest condition by reducing flow and heart rate to default values. Simulate with ‘Faster steady state’ option to reach resting steady state.

Use now ‘Adapt to Resting condition’.

…

[F]low (ml/s): 85

cycle [T]ime (ms): 850

…

Adapt n[O]ne [R]est,[E]xercise : **AdaptRest**

…

Wait for many beats, while adaptation takes place. The thus found state approximates the reference state sufficiently accurately for most applications. Accuracy may be increased by repeat of exercise and rest adaptation.

### Storing and retrieving a simulation

The reference simulation is stored as ParRef.mat, obtained by storing structure Par:

>>save ParRef Par

On may use any name for a mat-file, containing structure Par. The simulation may be retrieved by

>> CircAdaptMain, followed by selecting (**L**)oad.

The most compact way of storing a simulation is storage of the start condition only by execution of ‘Adapt0’, followed by storage of Par.

The most complete simulation with all time tracings is obtained by executing CircAdaptMain, followed by execution of CircAdaptDisplay, and storing Par in a mat.file. By retrieving this file, all time courses are ready for graphical display, e.g., aortic flow velocity is plotted as a function of time:

>> load ParXXX (mat-file containing Par)

>> figure(2); plot( Par.t, Par.ValveLArt.q ./ Par.ValveLArt.AOpen )

## Program in parts

The program is composed of a number of m-files, which will be briefly described below. Structure Par is globally accessible throughout most of the function in the program.

### CircAdaptMain

Script of main program to perform a CircAdapt simulation. Provides simple user interface.

### CircNew

Creates a Par-structure from scratch and executes **WriteSVarFiles**. Useful simulations require additional adaptation operations.

### WriteSVarFiles

Determines state variables in the Par-structure by searching for variables, ending with ‘Dot’, and writes files (**Par2SVar.m**, **Par2SVarDot.m**, **SVar2Par.m**) for communication between the Matlab ODE-solver (ode113) and the physiologic variables, stored in the Par-structure. Execution is absolutely needed after a change in the number and or identity of state variables.

### CircAdapt

The function CircAdapt calculates a simulation, based on initial conditions, as defined by state variables in structure ‘Par’. Many general conditions of the simulation are stored in ‘Par.General’. Execution occurs in steps of whole cycles.

### Timing

The function Timing determines the sequence of activation of all sarcomeres in all walls and patches of wall. Times of start of activation are stored in the relevant ‘Sarc’ structures.

### CircSVarDot

CircSVarDot calculates the set of time derivatives of state variables as a function of the set of state variables. To be used inside CircAdapt in combination with the Matlab ODE-solver.

### CircAdaptDisplay

Displays results of a simulation with state variables stored in ‘Par.SVar’. All signals are derived from available state variables, and stored in ‘Par’, having its largest size. Several hemodynamic variables are shown graphically in a figure.

### TubeDynamics

Calculates pressures and other variables of a tube from the tube volume, which is a state variable.

### CavityMech

The function CavityMech calculates cavity pressure for a given cavity volume. Calculations are performed in the following steps:

1) Mid-wall surface area is estimated. Areas of inlet and outlet channels (AmDead) have been subtracted to obtain midwall sheet area.

2) The function SheetMech converts mid-wall area to ‘membrane’ tension.

3) Within SheetMech, function SarcMech has been used to convert fiber strain to fiber stress.

4) Fiber strain is converted to tension.

5) Tension is converted to pressure.

### TriSeg

The function TriSeg is designed for two coupled cavities (left and right ventricle) to calculate two cavity pressures from two cavity volumes. Calculations have many steps in common with CavityMech with the addition that the balance of force between the three ventricular wall segments is used to calculate area and curvature of the walls.

### SheetMech

A sheet represents a curved wall segment. Given midwall area Am, curvature Cm, wall volume VWall and reference area AmRef, representative midwall tension T and transmural pressure pTrans are calculated (to be used inside CavityMech and TriSeg).

### SarcMech

Simulates the time dependent mechanical behavior of myocardial fiber. Natural fiber strain, as referred to sarcomere length LsRef, is used to calculate Cauchy fiber stress. SarcMech contains two state variables (unloaded sarcomere length Lsi and contractility C), which derivatives are also calculated.

### SackMech

In SackMech, for the pericardial sack, pressure is calculated as function of enclosed volume. During adaptation, maximum pressure is regulated by adaptation of the reference value enclosed volume ‘VRef’.

### SheetAdapt

For a sheet, wall volume, wall area and mean passive stiffness are adjusted by applying adaptation rules to hemodynamic data, available from the last beat.

### TubeAdapt

For a tube, diameter, wall thickness and pressure-volume working point are adjusted by application of adaptation rules to hemodynamic data, available from the last beat.

### Adapt0

Between beats, the last available state (SVar(end,:)) is used as starting condition for the next beat. Thus, the Par structure is reduced to one state in time only, causing a tremendous compression of Par for storage as a file.

### AdaptRest

Between beats, a selection of adaptation actions is performed for the resting state (diameter of blood vessels) .

### AdaptExc

Between beats, a selection of adaptation actions is performed for the state of exercise. (wall thickness of blood vessels and sheet geometry)

### WorkBalance

WorkBalance shows graphically to what extent work is conserved hemodynamically, on sheet mechanics and on fiber mechanics.

### MultiPatch *(inside SheetMech)*

Any wall (or Sheet) may be subdivided in patches, each having its own sarcomere with related state variables. If that condition occurs, for that wall MultiPatch will be executed. Thus in any wall, differences in activation time and other sarcomere properties can be simulated. This option is very useful to simulate pacing and infarctions. nSarc indicates the number of patches inside a sheet.

### SplitSheet

SplitSheet splits a given sheet in a chosen number of equal-sized patches, maintaining total wall volume and midwall area. (e.g. >> SplitSheet(‘Rv’,3);). Because the number of state variables increases, WriteSVarFiles is executed within the procedure to create and save new communication files. If the number of sheets is ≤1, the inverse procedure is carried out, i.e. a set of patches is replaced by a single sheet with average properties. After execution of SplitSheet, the information about the patches is stored in substructure 'Sarc'. Properties of the patches may be changed to wanted physiological properties.

### SteadyState

SteadyState is executed by ‘Faster steady state’-option in menu. It causes faster convergence to the steady state solution. This option is useful as long as its solution is stable. If it is not stable, switch off this option, and wait many beats until steady state is reached.

### .mat files

- Par.mat contains the state variables of the last simulation. For full availability of all variables within the Par-structure, CircAdaptDisplay has to be executed.

- ParRef.mat contains starting conditions of the reference simulation.

- ParExc.mat contains the start of the simulation of exercise.

- ParTemp.mat contains all data of the last successfully executed beat in a series of beats, to be carried out within function ‘CircAdapt’. The file ParTemp.mat is very useful for debugging purposes.
